# A fine scale eco-epidemiological study on endemic visceral leishmaniasis in north ethiopian villages

**DOI:** 10.1016/j.actatropica.2018.04.005

**Published:** 2018-07

**Authors:** Oscar David Kirstein, Laura Skrip, Ibrahim Abassi, Tamara Iungman, Ben Zion Horwitz, Araya Gebresilassie, Tatiana Spitzova, Yoni Waitz, Teshome Gebre-Michael, Petr Volf, Asrat Hailu, Alon Warburg

**Affiliations:** a Department of Microbiology and Molecular Genetics, The Institute of Medical Research, Israel-Canada [IMRIC], The Kuvin Centre for the Study of Infectious and Tropical Diseases, Faculty of Medicine, The Hebrew University of Jerusalem, Israel, Israel; b Department of Biostatistics, School of Public Health, Yale University,60 College street, New Haven, CT, 06520, USA, USA; c Aklilu Lemma Institute of Pathobiology, Addis Ababa University, Addis Ababa, Ethiopia, Ethiopia; d Dept. of Microbiology, Immunology and Parasitology, Faculty of Medicine, Addis Ababa University, Addis Ababa, Ethiopia, Ethiopia; e Department of Parasitology, Faculty of Science, Charles University, Vinicna 7, 128 44, Prague 2, Czech Republic, Czech Republic; f Department of Geography and Environmental Studies, Haifa University, Haifa, Israel, Israel

**Keywords:** Visceral leishmaniasis, Ethiopia, Cohort study, Ecoepidemiology, Phlebotomine sand flies, Vertisols

## Abstract

Visceral Leishmaniasis (VL) is a disseminated protozoan infection caused by *Leishmania donovani* that affects almost half a million people annually. In Northern Ethiopia, VL is common in migrant agricultural laborers returning from the lowland sesame fields of Metema and Humera. Recent VL foci have emerged in resident rural populations near the town. In the current study, we evaluate multilevel entomological, epidemiological and ecological factors associated with infection and disease through fine-scale eco-epidemiological analyses in three villages. Satellite images showed that villages constructed in or close to vertisols, were likely to become endemic for VL. Vertisols or black-cotton soil, are characterized by high contents of smectitic clay minerals, which swell when hydrated and shrink upon desiccation, causing extensive deep cracking during the dry season. The population densities of *Phlebotomus orientalis*, the vector, were negatively correlated with distance from vertisols and persons living close to vertisols were more likely to be bitten by sand flies, as evidenced by sero-positivity to *Ph. orientalis* saliva. Apparent (albeit non-significant) clustering of VL cases and abundant asymptomatic infections close to vertisols, suggest anthroponotic transmission around houses located close to vertisols. Comparable rates of male and female volunteers, mostly under 15 years of age, were infected with *L. donovani* but a significantly higher proportion of males succumbed to VL indicating a physiological gender-linked male susceptibility. Our data suggest that the abundant infected persons with high parasitemias who remain asymptomatic, may serve as reservoir hosts for anthroponotic transmission inside villages. Only limited insights on the transmission dynamics of *L. donovani* were gained by the study of environmental factors such as presence of animals, house structure and vegetation cover.

## Highlights

• We conducted fine-scale eco-epidemiological analyses of factors associated with visceral leishmaniasis transmission.
• The population densities of *Phlebotomus orientalis,* the vector, *were* negatively correlated with distance from vertisols.
• Sero-positivity to *Ph. orientalis* saliva, was found in people living close to vertisol areas.
• Apparent clustering of infections indicates that transmission occurs around houses located close to vertisols.
• Our data suggest that asymptomatic individuals serve as reservoir hosts for anthroponotic transmission inside villages.

## Graphical abstract

Hypothetical illustration of Anthroponotic Transmission Cycle of L. donovani in VL Endemic Villages in Northern Ethiopia. Transmission of *L. donovani* occurs anthroponotically in or close to households near to vertisols. Sand flies breeding in vertisols, are attracted into villages to feed blood on domestic animals, plant communities my serve as resting places for sand fly vectors.

**Figure:**
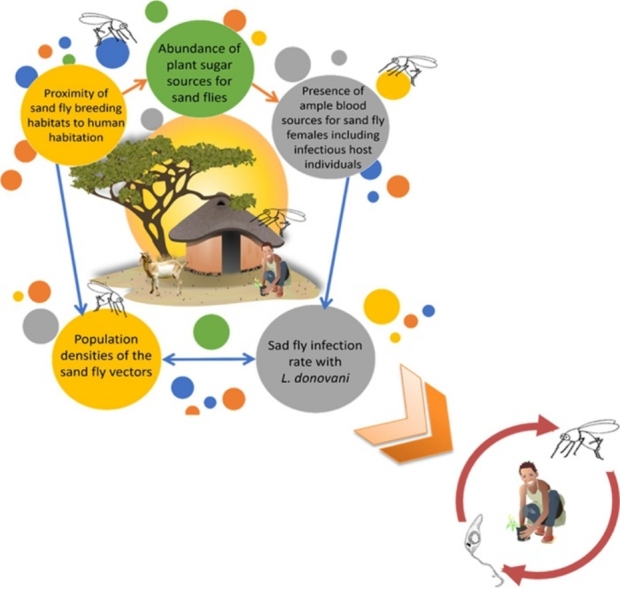

## 1 Introduction

Visceral leishmaniasis (VL), also known as Kala-Azar, is a vector-borne parasitic disease caused by protozoan parasites of the *Leishmania donovani* (Kinetoplastida: Trypanosomatidae) species complex and transmitted by blood-sucking phlebotomine sand flies (Diptera, Psychodidae). It is a systemic disease of the bone marrow, spleen and liver that primarily affects cells of the reticuloendothelial system (Alvar et al., 2012). VL is fatal in over 90% of the cases but timely medical intervention can boost survival rates by 80% (Guerin et al., 2002). Although molecular and serological methods can be indicative of disease, definitive diagnosis is complex and requires invasive medical procedures to obtain lymph node, spleen, or bone marrow aspirates for microscopical and/or molecular detection of *Leishmania* parasites.

VL is endemic in 98 countries with some 200 million people at risk, 200–500 thousand new cases and 20- 40 thousand fatalities annually (Alvar et al., 2012). Over 90% of the cases occur in six countries: India, Bangladesh, Nepal, Sudan, Ethiopia and Brazil (Chappuis et al., 2007). The species causing VL in the Indian subcontinent and East Africa is *L. donovani* donovani while *L.d. infantum* is the causative agent of VL in South America (Chappuis et al., 2007). In East Africa the worst affected countries are Sudan with an estimated 15–20 thousand cases and Ethiopia with 4,500–6,000 cases per year (Herrero et al., 2009) (Alvar et al., 2012). The worst affected regions in Ethiopia are the Humera-Metema lowlands bordering Sudan, which account for approximately 60% of the country’s reported VL cases (Leta et al., 2014). Emergence of VL in this region coincided with large-scale deforestation carried out to create vast, intensively cultivated farms producing mostly sesame, cotton and sorghum. The massive scale of these industrial agriculture enterprises necessitates a twice-yearly influx [and efflux] of some 500,000 seasonal workers among which, there is a high prevalence of HIV/VL coinfections (Alvar et al., 2012; Berhe et al., 2001; ter Horst et al., 2008).

Nineteen *Phlebotomus* species, representing six subgenera, have been documented in Ethiopia (Lewis, 1987). *Ph. martini* and *Ph. celiae* transmit VL in the more humid southern foci near the Kenyan border (Gebre-Michael and Lane, 1996) while *Ph. orientalis* is the predominant vector of VL in Northern Ethiopia and Sudan (Elnaiem, 2011; Gebre-Michael et al., 2004; Gebresilassie et al., 2015c).

**Fig. 1:**
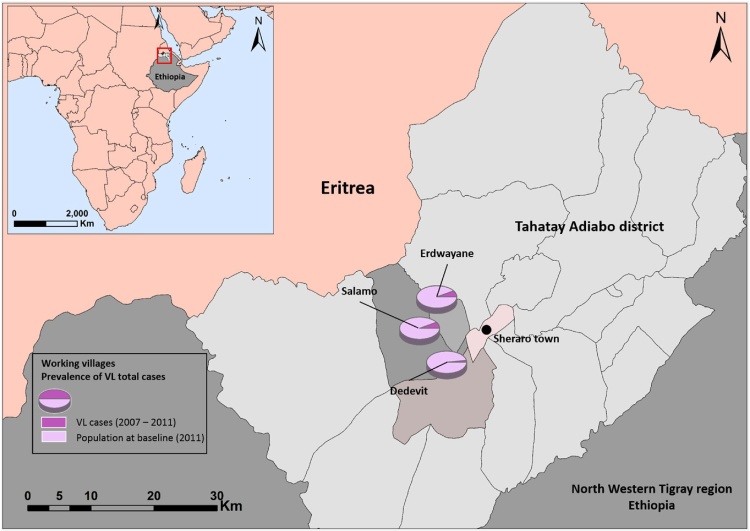
The Tahtay Adiabo District comprises the northern most tip of Ethiopia adjacent to the border with Eritrea (red frame in Inset). The district was selected due to high incidence of VL in rural villages close to Sheraro. Pie charts represent the total population at baseline (2011) and the fractional case incidence (2007–2015) in the three villages. (For interpretation of the references to colour in this figure legend, the reader is referred to the web version of this article.)

In 2006, a new focus of VL emerged in villages close to the town of Sheraro (District of Tahtay Adiyabo, northern Ethiopia, Fig. 1), probably as a result of infected seasonal laborers returning from the VL endemic areas of Humera and Metema (Herrero et al., 2009). Between 2006 and 2011, 209 VL cases and 3 PKDL were diagnosed and treated.

We initiated a multidisciplinary investigation of the Sheraro focus in 2009 performing both epidemiological and entomological studies (Abbasi et al., 2013; Gebresilassie et al., 2015c). The current study comprises part of this project. We hypothesized that transmission *L. donovani* depended on the population densities of *Ph. orientalis* vectors and the *L. donovani* infection rates among these flies. We further reasoned that, these parameters should be influenced by (1) The proximity of sand fly breeding and resting habitats (2) The presence of ample blood sources for sand fly females including infectious hosts. To address these questions we conducted a multilevel fine-scale eco-epidemiological investigation of entomological, epidemiological and ecological factors associated with the transmission of *L. donovani* to humans. Our analyses were founded in the disciplines of landscape-ecology and landscape-epidemiology that provide a conceptual framework for the study of vector-borne disease epidemiology (Emmanuel et al., 2011; Kitron, 1998; Reisen, 2010).

## 2 Materials and methods

### 2.1 Study area

Tahtay Adiyabo is one of 36 districts (woredas) in the Tigray Region of northern Ethiopia. The district is bordered to the south and south-west by Asigede Tsimbela and the Tekezé River, which separates Tahtay Adiyabo from Wolqayt and Setit Humera. On the north by Eritrea, and on the east by La'ilay Adiyabo. The administrative center of Tahtay Adiyabo is the town of Sheraro (14°23′41“N/37°46′15“E, 1246 m above sea level [masl]). The population of Tahtay Adiyabo is 90,144, with only 6377 (7.07%) living in towns. With an area of 3,841.51 Km^2^, Tahtay Adiyabo has a population density of 23.47 people/Km^2^, (2007 national census, Central Statistical Agency of Ethiopia) (Fig. 1).

### 2.2 Selection of the study villages

**Fig. 2:**
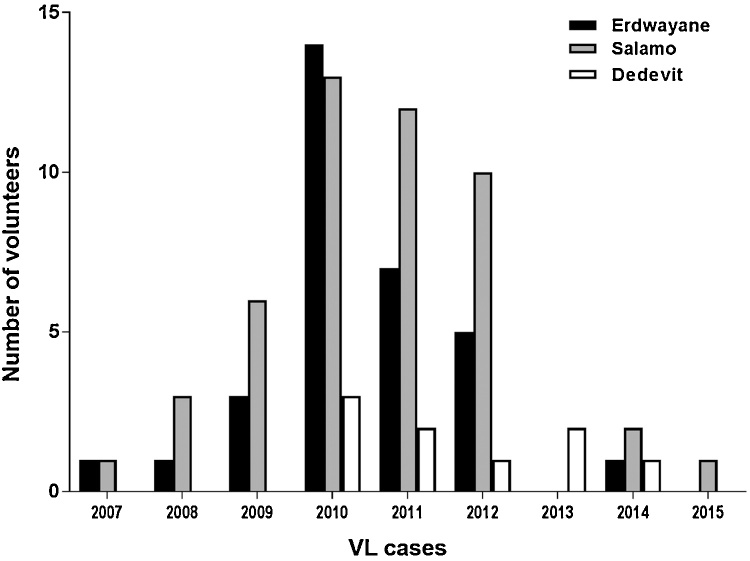
Visceral leishmaniasis (VL) cases in the three study villages. A total of 97 VL cases were reported, of these 49 had occurred before the study began (2007–2010) and 48 cases were reported while we were working there (2011–2015). The numbers of VL cases gradually declined from 2011 to 2015, at which time we concluded our studies.

In 2010 we conducted a census in the villages of Tahtay Adiyabo comprising data on 11,000 participants (personal data, socio-economic parameters, health status and clinical history of VL). Based on this census, 18 villages were selected for the cohort studies (Abbasi et al., 2013). The three villages included in the current study were selected based on data from the above census and cohort studies, including the percentage of VL cases; two of the villages (Salamo and Erdwayane) had high incidence of VL and one (Dedevit) had very low incidence (Fig. 2). Baseline parameters such as sand fly species richness and abundance, environmental and geographical variables, accessibility to motorized transportation as well as other logistic hurdles, were also taken in consideration. The three villages cover an area of 7.80 km^2^ combined and comprise 367 households, with a total population of 1540 inhabitants. Average household size is 4–5 persons and the average age of the houses is 7.5 years for Erdwayane, 12.3 years for Salamo, and 16.8 years for Dedevit. The population density in Erdwayane was 414 persons/km^2^, in Salamo 99/km^2^, and in Dedevit it was 272 persons/km^2^.

The villages are built on sand-stone hills and outcrops surrounded by vertisol fields, which are used mainly for agricultural cultivation and pasture. The landscape is distinct in the three villages. Salamo and Erdwayane are more rural with open spaces and traditional houses whereas Dedevit is more urbanized with asphalt roads and houses connected to the electric grid. The hills upon which the villages are built are largely deforested due to woodcutting for construction and cooking. The remaining vegetation comprises scattered copses of *Acacia* sp., *Balanites* sp., and *Zyziphus* sp. trees/bushes. The introduced Neem tree (*Azadirachta indica*) is also abundant around homesteads.

### 2.3 Ethical considerations

Informed consent was obtained from all the adult volunteers who participated in the study. Consent for inclusion of young children, was obtained from parents or guardians. The study protocols were reviewed and approved by the ethical review committee at the Medical Faculty, Addis Ababa University and the National Research Ethics Review Committee (NRERC) at the Ethiopian Ministry of Science and Technology. Each volunteer was treated in accordance with international bio-ethical rules and laws for human sampling. Blood was sampled by certified trained Health officers or nurses. Volunteers requiring medical assistance were treated by project MDs or transported to regional hospitals.

### 2.4 Research design

A cohort study was conducted in the three villages. Volunteers were recruited based on the historical roster of all those previously screened. Former VL Cases were defined as clinically confirmed cases. During the study itself, persons presenting with fever lasting 2 weeks or longer, weight loss and/or splenomegaly and diagnosed either parasitologically or serologically (rk39 dipstick) were considered current VL cases.

To record the data we used an interactive tablet-based poll application QuickTapSurvey (http://www.quicktapsurvey.com Toronto, Canada, 2013). Cases and controls responded to structured interviews summarizing demographic and socio economic characteristics such as age, gender, occupation, education, sleeping habits, bed net use, family size, period living in the current location/house, house construction (materials and condition), ownership of domestic animals (species and number) and farming activities. Respondents were interviewed by trained health professionals in their indigenous languages (Amarignia, Tigrigna and/or Kunamigna).

### 2.5 Blood sampling

Asymptomatic infections were diagnosed by PCR that detected Leishmania DNA in blood as the best indication of infection (Singh et al., 2014). Blood samples were obtained by (i) Finger pricks using disposable blood lancets. (ii) Venous blood was collected using the BD Vacutainer collection system with serum-separating tubes (Gold cap BD Vacutainer^®^ tubes SST ™ with BD Hemogard™ closure). To identify parasite DNA, four drops of finger prick or venous blood were blotted on Whatman No1 filter papers and kept dry. For serology, tubes were centrifuged at 13,000 rpm for 15 min, then, sera were pulled, aliquoted and stored at −80 °C.

### 2.6 DNA extraction and PCR amplification

DNA was extracted with phenol-chloroform from two paper punch disks (r = 3 mm), of Whatman No1 filter paper calculated to have been saturated with approximately 20 μl of blood each, and tested for *L. donovani* infection via quantitative kDNA real-time PCR (qRT-kDNA/PCR) as previously described (Abbasi et al., 2013). Samples that tested positive were assessed via conventional PCR to amplify and sequence a *Leishmania* species-specific segment of the internal transcribed spacer 1 (ITS1) (El Tai et al., 2000).

### 2.7 Anti-*Leishmania donovani* IgG serology

#### 2.7.1 Antigen preparation

Antigen was prepared from a virulent Sudanese *L. donovani* isolate (MHOM/SD/??/Khartoum). Promastigotes were cultured in M199 medium containing 10% fetal calf serum and antibiotics (Debrabant et al., 2004). 10 ml of 5 × 10^7^/ml promastigote culture were centrifuged at 4000 rpm for 10 min, and washed twice with phosphate buffered saline (PBS: 155 mM NaCl, 160 mM Na_2_HPO_4_, 4 mM KH_2_PO_4_; pH 7.4). The parasite pellet was resuspended in TE lysis buffer (40 mM sodium chloride, 10 mM Ethylene Diamine-tetra-acetic acid [EDTA, pH 8], 50 mM Tris-HCl, pH 7.5 and 1X Protease Inhibitor Cocktail [Sigma-Aldrich, St. Louis, USA]), sonicated for 15 min in an ultrasonic bath and chilled in an ice bath for 20 min. The lysate was centrifuged for 10 min at 3000 rpm and the supernatant was decanted, aliquoted and stored at −70 °C.

#### 2.7.2 Enzyme-Linked-Immune sorbent assay (ELISA) for determining anti- *L. donovani* antibody titers

Wells, (Nunc-Immuno™ MicroWell™ 96, Thermo Scientific, Denmark) were coated with *L. donovani* promastigote lysate (10 μg protein per well) in 20 mM carbonate-bicarbonate buffer (pH 9.25) overnight at 4 °C, washed in PBS and incubated with 200 μl/well blocking solution (5% fetal calf serum, 0.05% Tween-20 and PBS pH7.2) for 30 min at 37 °C. Volunteer sera samples (diluted 1:200 and 1:400 in the same blocking buffer − 200 μl/well) were incubated in duplicate for two hours at 37 °C. Thereafter, Protein-A conjugated Horseradish Peroxidase (HRP, Jackson ImmunoResearch Laboratories, INC. PA, USA) conjugated secondary antibodies were added and plates were incubated for 1 h at 37 °C. The plates were rinsed with PBS-T (155 mM NaCl, 160 mM Na_2_HPO_4_, 4 mM KH_2_PO_4_; pH 7.4, and 0.05% Tween) followed by addition of ABTS substrate (Sigma-Aldrich, St. Louis, USA). The absorbance at 405–190 nm was measured in a SPECTROstar^®^ Nano microplate reader (BMG LABTECH, Germany).

### 2.8 Enzyme-Linked-Immune sorbent assay (ELISA) for anti-*Ph. orientalis* saliva antibodies

To estimate the exposure of volunteers to *Ph. orientalis* bites, anti-saliva IgG antibodies in volunteers' sera were measured via ELISA. Microtiter plate wells (Nunc-Immuno™ MicroWell™ 96, Thermo Scientific, Denmark) were coated with *Ph. orientalis* salivary gland homogenate (SGH, corresponding to 0.2 gland/well, prepared as previously described (Vlkova et al., 2011) and diluted in coating buffer (Carbonate-Bicarbonate, pH 9) and incubated overnight at 4 °C. The wells were then washed with 100 μl of PBS-T and incubated with 100 μl of blocking/diluting solution (2% [w/v] low fat dry milk in PBS-T) for 60 min at 37 °C to block free binding sites. Thereafter, sera were diluted 1:50 and 1:100 in diluting solution/coating buffer and incubated in duplicate for 90 min at 37 °C. Secondary antibody, α-human IgG HRP (Sigma) diluted 1:1000 was added in a total of 100 μl and incubated for 45 min at 37 °C. Lastly the unbound antibodies were washed six times using PBS-T three minutes per wash. 100 μl of orthophenylenediamine and H_2_O_2_ in McIlwain phosphate-citrate buffer (pH 5.5) were used as a substrate solution. After 5 min incubation in the dark the reaction was stopped by adding of 100 μl of 10% H_2_SO_4_. Absorbance was measured with a Multiskan RC ELISA reader (Labsystems) at 492 nm wavelength.

### 2.9 Entomology

#### 2.9.1 Trapping methods and sand fly surveillance

Sand fly traps were deployed in trapping units (TUs), comprising one CDC light trap (6 V, 150 mA, John W. Hock, Model 512, Gainesville, FL) and two large Sticky Traps (ST) made of white polypropylene boards, measuring 60 × 60 cm, placed horizontally on a square metal frame supporting them approximately 15 cm above ground, with the upper face of the board smeared with sesame oil (Moncaz et al., 2013). The three traps comprising the TU were positioned in a triangular arrangement, 2–3 m apart and deployed either inside the domestic compound or in peridomestic areas. Sand flies were trapped during dry season (March to April 2013). 13 TU were deployed in the village of Salamo, 14 in Erdwayane and 12 in Dedevit (Table 2). TUs operated between 6:00 PM to 6:00 AM the following morning. Sand flies collected by the three traps in each TU were pooled for statistical analyses.

Sand flies were preserved in 100% EtOH and transported to the laboratory for taxonomical identification. All specimens belonging to the genus *Phlebotomus* and about 5–10% of the *Sergentomyia* spp. were selected for species identification under a stereo microscope. To identify species, the heads of males and females were removed to expose the internal structure of the pharynx and cibarium. In females, the end of the abdomen was dissected as well, to expose the spermatheca. Flies were mounted in Hoyer’s medium on microscope slides. Identification of species was based on cibarial, pharyngeal armature, antennal features and spermathecae of females and external genitalia of males, using different keys (Artemiev, 1980; Kirk and Lewis, 1946; Lewis, 1982; Perfil'ev et al., 1968; Quate, 1964).

#### 2.9.2 Nocturnal activity rhythms

A Collection Bottle Rotator (CBR – John W. Hock. Model 1512) was used to separate the nightly catch into 8 different bottles corresponding to a predetermined period using a programmable timer. The timer was set to rotate every 1.5 h, from 18:00 in the evening until 7:30 the following morning. CBRs were deployed in two different ecotopes (A- Farm field and B – Peridomestic), for 6 consecutive nights, during April 2013.

#### 2.9.3 DNA extraction from sand flies

Prior to dissection, sand flies were placed in a tea strainer, rinsed under tap water, briefly immersed in dilute dish-washing detergent, rinsed again, immersed in 1% sodium hypochlorite (NaClO) solution for 1 min and rinsed in double distilled water to remove all external contamination. DNA was extracted from individual sand flies using phenol-chloroform as previously described (Abbasi et al., 2009)

#### 2.9.4 *Leishmania* infection in sand flies

*Leishmania* infection rates were determined by qRT-kDNA/PCR as described above for blood samples (Abbasi et al., 2013). Sand flies were pooled in pools of 6 specimens per reaction. Positive samples were re-amplified by ITS1 PCR and amplicons were sent for DNA sequencing to identify the *Leishmania* species (El Tai et al., 2000). Validation of parasite species (i.e. *L. donovani*) was based on ITS1 sharing >99% sequence homology with existing *L. donovani* entries in GenBank.

#### 2.9.5 Determination of sand fly blood sources by Reverse Line Blotting (RLB)

Blood hosts were identified as previously described (Abbasi et al., 2009). Briefly, DNA extracted from abdomens of blood fed *Ph. orientalis* females (and a small number of other *Phlebotomus spp.*) was PCR amplified using biotinylated vertebrate species-specific cytochrome b primers. The PCR products were blotted on species-specific oligonucleotides. Specific hybridization was visualized using streptavidin horseradish peroxidase.

### 2.10 DNA sequence analysis

PCR amplicons (from human blood samples and sand flies) were sequenced by The Center for Genomic Technologies at the Hebrew University of Jerusalem using automated DNA Sequencing, based on BigDye Terminator cycle sequencing chemistry from Applied Biosystems (ABI), ABI PRISM 3730xl DNA Analyzer and the ABI’s Data collection and Sequence Analysis software. The derived sequences were compared against the GenBank database using NCBI BLAST (https://blast.ncbi.nlm.nih.gov/Blast.cgi) and default search parameters (Madden, 2013).

### 2.11 Meteorological data

Data loggers with sensors (HOBO Mini-Weather Station, Onset Computer Corporation, Pocasset, MA, and U.S.A) were used to record temperatures (°C) and relative humidity (RH%) during experiments.

### 2.12 Statistical analysis

#### 2.12.1 Analysis of entomological data

Abundance of sand flies including *Ph. orientalis* was calculated based on the number of flies captured per trapping unit (see above item 9.1) per night. Data was tested for its distribution and variance by 1-Sample Kolmogorov – Smirnov Z test (K-S normality test) and Shapiro–Wilk test. The nonparametric tests Kruskal–Wallis (K-W) and Mann–Whitney-U (M-W) were applied to analyze non-transformed data. K-W test was employed to compare the median number of sand flies in general or *Ph. orientalis* specifically collected in the different villages. When K-W was significant Dunn’s test for pairwise multiple comparisons was followed. P value was adjusted with Bonferroni correction.

Nocturnal activity, One-way ANOVA was used to compare the hourly activity patterns of male and female *Ph. orientalis* during the night. Tukey’s Studentized test post hoc analysis was utilized for mean separation where ANOVA was significant. For the same analysis, Spearman’s rank-correlation analysis (*P* < 0.05) was also used to compare the effects of average nighttime temperature and humidity on the number of flies captured per hour.

For each categorical variable, the distribution for frequencies across villages was analyzed using a chi-square or Fisher’s exact test depending on whether counts were more or less than five, respectively. Pairwise analyses using adjustment by the Benjamini–Hochberg method (i.e., false discovery rate) were conducted for those variables with significant results in the universal test. Continuous variables were described in terms of means and standard deviations.

For the logistic regression models, PCR positivity was defined as ≥1 parasites per ml blood at baseline measurement and clinical VL was defined as developing disease at any time during the study. Unadjusted analyses evaluating the associations between outcome variables and individual-households, were conducted for each village separately using logistic regression.

Multilevel analysis was conducted for data from the entire study sample in aggregate, using a generalized linear mixed model fit by the Laplace approximation.

Statistical analyses were performed using IBM SPSS Statistics for Windows Version 23.0 (IBM CorPh., Armonk, NY, U.S.A.), in Microsoft^®^ Office Excel 2013 (Microsoft CorPh., Redmond, WA, U.S.A.) and R version 3.4.0 (R Foundation for Statistical Computing, Vienna, Austria). Results were considered significant when P < 0.05, unless stated otherwise.

#### 2.12.2 Spatial analysis

Integration of Geographic information systems (GIS) and Remote Sensing (RS) was used to allow the digitalization and projection of epidemiological and entomological data and permit mapping and evaluation of environmental factors related to the spatial and temporal distribution of the VL cases as related to vector distribution.

Spatial data included: the location of houses, land use and land cover as well as the position of the TUs. Data were recorded as waypoints using a GPS device (Garmin *Dakota 20*), with an accuracy-range of 4–5 m. GPS data were downloaded, projected, codified and rectified into ExpertGPS Map software 5.20 for GARMIN devices (TopoGrafix).

Local distribution maps that depict “hot-spots”, areas characterized by high abundance of sand flies and people seropositive for sand fly saliva, were generated using the Inverse Distance Weighting (IDW) interpolation method. Numbers of *Ph. orientalis* per TU per night and the ODs (optical densities) values of anti-*Ph. orientalis* saliva obtained by ELISAs were represented on these maps.

Linear regression was applied to evaluate association between the estimated number of *Ph. orientalis* per TU (as the dependent variable) and the estimated ODs anti-*Ph. orientalis* saliva ELISAs (as the independent variable). This analysis was done for populated areas only. We represented peridomestic areas as a circular buffer zone around each household (r = 100 m). Furthermore, masks were issued from the results of the produced surface maps to obtain two continuous data sets. The association between the distribution of sand flies and the exposure of people to their saliva and the distance from vertisols was calculated as the shortest Euclidean distance from data collection point (TU or house of exposed volunteer) to the vertisols area. A linear regression analysis was performed to test for association between the number of *Ph. orientalis*/per TU (as the dependent variable) and the distance from vertisols (as the independent variable). In case of non-linear relationship between the variables, the variable distance was Ln transformed to enable linearization of the variables in the model. For GIS spatial analysis vertisol layers were demarcated by manually generated polygons.

#### 2.12.3 Remote sensing

To study the areas covered by vertisols and their dynamic seasonal changes, we used remotely sensed satellite images of the Sheraro area (NW corner: 15°26′40.20“N, 36°44′32.35“E; SW corner: 38°28′28.42“E, 13°27′34.52“N). Satellite images spanning September 29, 2013 (rainy season, Fig. 7A), January 16, 2014 (early dry season, Fig. 7B), and April 19, 2014 (late dry season, Fig. 7.C) were acquired from Landsat 8 −OLI dataset, Earth Explorer (USGS, http://earthexplorer.usgs.gov/). Supervised classification method was used to cluster pixels in the digital scene dataset into desired spectral classes. Regions of interest (ROI) were generated as defined training classes in the output images corresponding to vertisol areas. Afterwards, maximum likelihood classification was performed to assign to each pixel in the subset image data the trained class of which it had the highest probability of being a member, in this case of being vertisols (Exelis Visual Information Solutions, 2014; Sithiprasasna et al., 2005). The analyses employed data processing based on the following bands: Band 2 (Blue: 0.452–0.512 μm), Band 3 (Green: 0.533– 0.590 μm), Band 4 (Red: 0.636–0.673 μm) with 30 m spectral resolution.

Analyses were carried out using ArcGIS™ 10 (ESRI Inc. Redlands, CA, USA) and ENVI (Exelis Visual Information Solutions, Boulder, Colorado).

## 3 Results

### 3.1 Epidemiological data: characteristics at the level of the individual

Data was collected from 1127 of 1540 individuals residing in the three villages. The majority of the participants were female (598/1127; 53.0%), and under 15 years of age (314/598; 52.5%). The mean coverage during the four cohort samplings exceeded 73.2% of the populations [Erdwayane: 77% (354/460); Salamo: 60% (357/596) and Dedevit: 86% (416/484)]. Ninety seven clinical VL cases were recorded, 45 had contracted VL prior to our study (2007–2010) and 52 during the follow up period of the cohort (2011–2015) (Table 1). The numbers of VL cases gradually declined beginning 2011 but remained endemic until 2015 (Fig. 2).

**Table 1:** Incidence of visceral leishmaniasis and asymptomatic infections with *Leishmania donovani* in the three experimental villages (determined in 2011 prior to the beginning of the current study).

Individuals (sampled/total population)^a^	Overall (1127/1540)^a^	Erdwayane (354/460)	Salamo (357/596)	Dedevit (416/484)	P-value*
PCR positivity (1–9 parasites per ml blood)^b^	198 (17.5%)	50 (14.1%)	81 (22.6%)	67 (16.1%)	<0.001
PCR positivity (>10 parasites per ml blood)^b^	77 (6.8%)	9 (2.5%)	57 (15.9%)	11 (2.6%)	<0.001
Total PCR positivity^b^	275 (24.4%)	59 (16.6%)	138 (38.6%)	78 (18.7%)	<0.001
Age (mean ± SD)	20.2 ± 17.6	20.3 ± 19.2	17.7 ± 14.9**	23.0 ± 18.4**	< 0.001
VL cases (2007–2015)^c^	97 (6.3%)	40 (8.7%)	48 (8.1%)	9 (1.8%)	<0.001
VL cases (2011–2015)^c^	52 (3.4%)	21 (4.5%)	25 (4.1%)	6 (1.2%)	<0.001
Female Sex	802 (52.0%)	234 (50.8%)	304 (51.0%)	263 (54.3%)	
Male sex	738 (47.9%)	226 (49.1%)	292 (48.9%)	220 (45.4%)	0.473

* Significantly different by Chi-square post hoc test.

** Significantly different by Tucky’s HSD test post hoc analysis.

a The values in () on the first row are sample/total population as is indicated.

b Percentages are calculated upon the sampled population.

c Percentages are calculated upon the total population.

**Table 2:** Total numbers + medians and interquartile intervals of sand flies caught near households. Numbers represent sand flies/TU/night captured in the three experimental villages (2013).

	Erdwayane (n* = 13)		Salamo (n* = 14)		Dedevit (n* = 12)		Overall	
	No.	Median (IQI)*	No.	Median (IQI)	No.	Median (IQI)	No.	Median (IQI)
Sand flies	8642	194.5 (154.0, 355.3)	4531	126.2 (111.0, 183.5)	6504	149.5 (111.5, 313.6)	19,677	158 (111.5, 313.7)
Sergentomyia spp.	7120	1.3 (0.5, 2.5)a,b**	3919	119.9 (107.7, 146.7)a**	6155	139.0 (97.8, 205.3)b**	17,194	105.3 (2.4, 136.7)**
*Phlebotomus spp*	1522	19.5 (12.0, 76.0)	612	6.9 (2.7, 21.7)	349	5.7 (4.3, 14.1)	2483	12.0 (4.6, 28.8)*
*Phlebotomus orientalis*	1469	19.5 (11.3, 73.0)a*	566	4.4 (1.3, 21.3)	296	3.5 (1.8, 13.2)a*	2331	11.3 (2.2, 21.7)*
*P. orientalis* males	854	11 (5.0, 40.5)a*	415	2.7 (1.0, 13.3)	175	1.3 (0.1, 7.9)a*	1444	4.5 (1.0, 16.5)*
*P. orientalis* Females	615	8.5 (2.7, 20.5)a*	151	1.8 (0.3, 8.0)a*	121	2.4 (1.3, 5.2)	887	2.7 (1.3, 11.2)*
*L. donovani* infection rates in *P. orientalis* females	0.027 (17/615)		0.23 (35/151)		0.03 (5/212)			

Values followed by letters are significantly different between villages (Dunn's nonparametric comparison post hoc for K-W test. P values were adjusted with Bonferroni correction).

^*^Asterisks following letters, show the level of significance by K-W Test.

Data if presented as Medians (IQR – Interquartile Intervals). Medians, were calculated from the Means of sand flies per TU per night.

During the course of the study (2011–2015) higher incidence of VL was reported in Salamo (4.1%, 25 VL cases) and Erdwayane (4.5%, 21 VL cases). Whereas incidence in Dedevit was much lower (1.2%, 6 VL cases) (Table 1). Out of the total number of reported VL cases, 65.3% (34/52) were individuals under 15 years. In general, more males developed disease outcomes than females [69.2% (36/52) and 30.7% (16/52) respectively].

**Fig. 3:**
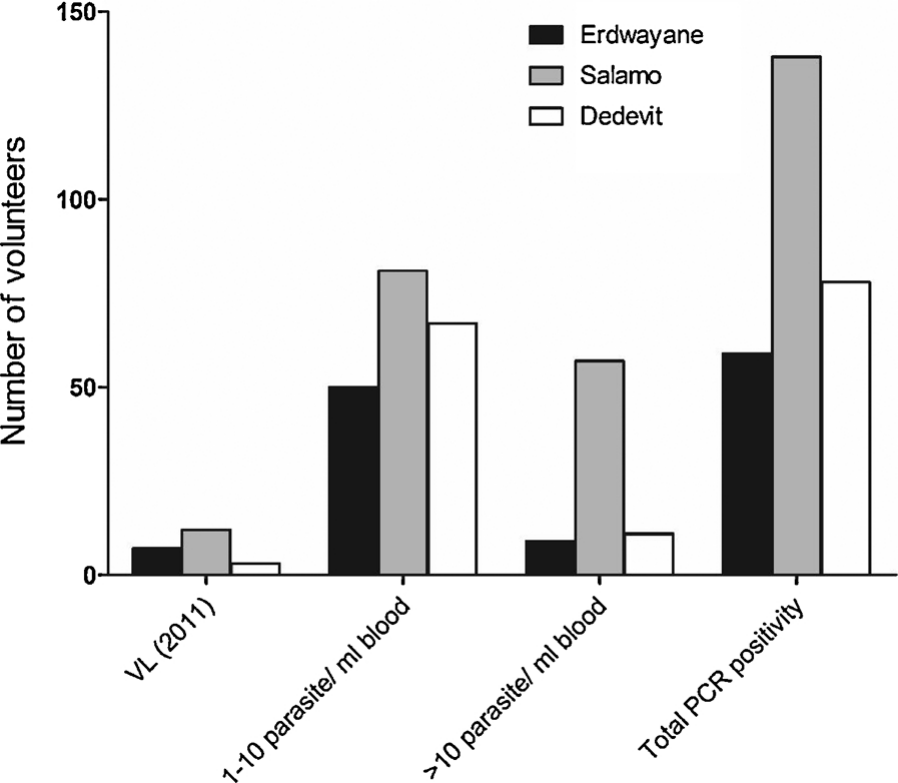
VL cases and asymptomatic infections at baseline sampling (2011). Most of the asymptomatically infected (qRT-kDNA/PCR positive) population scored 1–10 parasites per ml blood (17.5%), and 6.8% had >10. A significantly higher number of PCR positive volunteers was found in the village of Salamo (χ^2^ test = P < 0.001).

Tested by qRT-kDNA/PCR, 17.5% (198/1127) of the sampled population were asymptomatically infected with 1–10 parasites per ml blood while 6.8% (77/1127) had >10 parasites per ml blood. High parasitemias (≥100 parasites per ml blood) were found in 3.5% of the population (40/1127). A significantly higher number of asymptomatic infections was found in the village of Salamo (χ2 test = P < 0.001), and the rate of asymptomatic individuals with high parasitemias was highest in Salamo too (57/357, 15.9%) (χ^2^ test = P < 0.001) (Table 1, Fig. 3). Although a higher proportion of males developed VL (69% (36/52), we found that slightly more females (25% (16/52) than males were asymptomatically infected (13.3% and 10.9% respectively). Infection intensities did not differ significantly between males and females (χ^2^ test = P > 0.05).

Among those individuals that retrospectively reported clinical VL (contracted before 2011) for whom PCR data were available, 6/49 (12.2%) remained PCR positive during the initial sampling in 2011 (3 patients with 1–10 and 3 with >10 parasites per ml blood). Similarly, 4/48 (8.3%) individuals who developed clinical disease during the study period remained PCR positive 2–3 years later including the final sampling in 2015. Remarkably three VL cases reported before 2011, presented low parasitemia (≥1 parasites per ml blood), and remained PCR positive until the final sampling in 2015. The overall ratio of symptomatic to asymptomatic infected individuals (at any parasitemia and at baseline) across the three villages was calculated as 1:9.

A limited number of volunteers who participated in the past cohort studies was sampled again in 2015 in two of the three villages, Salamo and Dedevit. Blood samples were obtained from 126 volunteers via finger-pricks for PCR. Serum was separated from venous blood for serology. Of the total number of individuals sampled, 44 were past VL cases recorded as of 2007 (VL treated cases), and 82 individuals never had VL (healthy negative) (Table 1 Supp. data).

Anti- *L. donovani* antibodies were detected in 85 (67.4%) individuals, 40 (31.7%) of whom were past VL cases and 45 (35.7%) healthy individuals without a history of VL. *L. donovani* DNA was detected in 35(27.7%) volunteers, 20 (15.8%) of which were past VL cases, and 10 (7.9%) healthy negative individuals. Thirty of the volunteers were concurrently positive by ELISA (anti-*L. donovani* Abs) and PCR (*Leishmania* DNA). Of these 20 were past VL cases (11 of them had >10 parasite per ml) and 10 were healthy (of those 5 volunteers with parasitemia of >10 parasite per ml and 2 presented parasitemia of >100 parasite per ml). Some 20 (15.8%) past VL cases and 35 (27.7%) healthy volunteers were seropositive by ELISA but negative by PCR (Table 1 Supp. data). One past VL case (0.7%) was seronegative but PCR positive, and 3 (2.3%) were negative for both parameters. Likewise, 4 (3.1%) healthy individuals were positive for k-DNA but seronegative and 33 (26.1%) were negative for both parameters (Table 1 Supp. data). Past VL cases from Dedevit had relatively high titters of anti-*L. donovani* antibodies (ELISA down to 1:1600 serum dilution). We assume this was the case because all VL cases from Dedevit were relatively recent (since beginning of 2010). In Salamo, one individual that was treated during 2008, still presented with high titters of anti-*L. donovani* Abs during of the healthy seropositive individuals remained ELISA positive at dilutions downto1:1600.

### 3.2 Entomology

#### 3.2.1 Sand fly bionomics

Sand fly collections were conducted from early February to late May in 2013/14. Seven *Phlebotomus* spp. and 10 *Sergentomyia* spp. were identified (Table 2 Supplementary data). The overall population densities of all species of sand flies did not differ significantly between the three villages (Kruskal Wallis test χ2 (2) = 3.027, P = .220). The median TU catch was 158 (111.5, 313.7)/TU/night. The vast majority of sand flies (87.3%) were *Sergentomyia spp*. All 2483 *Phlebotomus* spp. were identified showing that *Ph. orientalis* was by far the predominant species in the genus (93.9%, Table 2 and Table 2 Supplementary data). The male/female ratio of *Ph. orientalis* was consistently and significantly male biased (males/females (1444/887 = 1.62; Mann Whitney *U* test, U = 4,192.5, P = .465; Table 2).

Significantly different numbers of *Ph. orientalis* were recorded in the three villages (Kruskal Wallis test χ2 (2) = 6.417, P = .040) with high numbers in Erdwayane, intermediate in Salamo and the lowest numbers in Dedevit (Table 2)

#### 3.2.2 Nocturnal activity of *Phlebotomus orientalis*

**Fig. 4:**
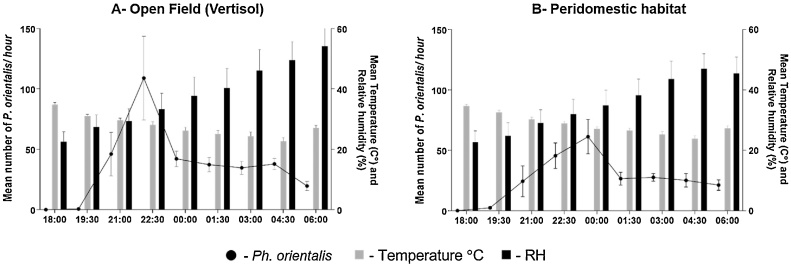
Nocturnal activity of *Phlebotomus orientalis* at different hours of the night and average temperature and relative humidity values. (A) A fallow vertisol field. (B) A peridomestic village setting. Trapping was conducted during late march 2013, using Bottle Rotator Traps (BRTs). Note the lag of about 90 min between the population peak in the open field (A, 21:00-22:30) and the village (B, 22:30–12:00 pm).

Nocturnal activity patterns of *Ph. orientalis* were studied in Erdwayane, where two Bottle Rotator Traps (BRT) were used. In total, 13,348 sand flies were collected by two BRTs, one placed in an open vertisol field (Fig. 4A, n = 8786) and the second one in a peridomestic area (Fig. 4B, n = 4562). Sand flies were separated into *Sergentomyia* spp. (A = 6494 [73.9%] and B = 2923 [64.0%]) and *Ph. orientalis* (A = 2293 [26.0%] and B = 1639 [35.6%]). Fewer females than males were captured throughout the night (male/female ratio = 1.5 in both locations).

The densities of *Ph. orientalis* populations were significantly different during collection intervals (ANOVA, F (df = 8) = 2.75; P < 0.000). A peak in nocturnal activity (108.9 flies/trap/hr) occurred between 21:00-22:30 h in the open field (Fig. 4A), and somewhat later between 22:30-00:00 in the peridomestic area 61.33 flies/trap/hr, Fig. 4B).

#### 3.2.3 *L. donovani* infection rates in *Phlebotomus orientalis*

*Ph. orientalis* females (978) were screened for *Leishmania* infection by qRT-kDNA/PCR. Parasite species was confirmed as *L. donovani* by sequencing of the ITS1 amplicons in 57 samples. No other *Leishmania* spp. were identified in any of the kDNA positive *Ph. orientalis*. The highest rate of *L. donovani* infections was found in *Ph. orientalis* from Salamo (23%), followed by Dedevit and Erdwayane with 3 and 2.7% respectively (Table 2).

#### 3.2.4 Host feeding preferences of *Phlebotomus orientalis*

The source of blood meals ingested by 150 female *Ph. orientalis* (50 samples from each village) was identified using *cyt b* PCR followed by vertebrate *cyt b* specific reverse line blotting (RLB). A high proportion *Ph.* orientalis was found to have fed only on bovine (79, 52%) or human (22, 14.7%) blood. Forty-nine (32.7%) of the blood-fed flies had mixed blood meals. Of these, the majority were bovine/human (34, 22.7%). Other blood sources were also detected, however always as mixed blood meals (e.g. camelid/bovine avian/human, canine/bovine.

### 3.3 Integrated analysis of variables potentially relevant to transmission of *L. donovani*

#### 3.3.1 Household characteristics

Across the three study villages, individuals from 324/367 households were included in the analysis. Most of the compounds had a shelter outside the house (Dase) and/or an animal shed (Dambe) (n = 241, 75.3%; n = 201, 62.8%, respectively), with no significant variation across the three villages. Households in Dedevit were less likely to have any animal than those in Erdwayane and Salamo (adjusted *p*-values: 0.028 for both locations), and more likely to have tin roofs (adjusted *p*-values: < 0.001 for both locations). Fewer households in Salamo had stone fences compared with households in Erdwayane and Dedevit (adjusted *p*-values: 0.002 and <0.001, respectively) (Table 3).

**Table 3:** Study population characteristics at the household-level.

Households (Samples/total)	Overall (n = 324/367)	Erdwayane (n = 92/110)	Salamo (n = 113/124)	Dedevit (n = 119/133)			P-value*
Participants per household	4.8 ± 2.4	5.0 ± 2.7	5.3 ± 2.2		4.1 ± 2.2		
At least one PCR+ household member (1–9 parasites per ml blood)	160 (49.4%)	39 (42.4%)	74 (65.5%)		47 (39.5%)	<0.001	
At least one PCR+ household member (>10 parasites per ml blood)	55 (17.0%)	8 (8.7%)	38 (33.6%)		9 (7.6%)	<0.001	
Dase (Resting shelter)	241 (75.3%)	64 (71.1%)	84 (74.3%)		93 (79.5%)	0.366	
**Dambe (Animal shed)**						0.177	
None	119 (37.2%)	43 (47.8%)	36 (31.9%)		40 (34.2%)		
Inside compound	159 (49.7%)	37 (41.1%)	60 (53.1%)		62 (53.0%)		
Outside compound	42 (13.1%)	10 (11.1%)	17 (15.0%)		15 (12.8%)		
Stone fence	220 (68.8%)	56 (62.2%)	47 (41.6%)		117 (100.0%)	<0.001	
Tin roofed house	270 (84.4%)	75 (83.3%)	79 (69.9%)		116 (99.1%)	<0.001	
Any animal (camel, donkey, goat, sheep, dog, cat)	80 (35.7%)	28 (40.6%)	33 (56.9%)		19 (19.6%)	<0.001	

Number of households (versus number of individuals).

* Using the Tukey’s post hoc test, Dedevit tended to differ from the other two villages.

Nearly 50% of the sampled households had at least one resident testing PCR positive (≥1 parasites/ml: n = 160, 49.4%). Fifty five (17%) volunteers had higher parasitemias >10 parasites per ml blood. Households in Salamo were significantly more likely to have at least one person with parasitemia than those in both Erdwayane (adjusted *p*-value for 1–10 parasites/ml: 0.002; adjusted *p*-value for >10 parasites/ml: <0.001) and Dedevit (adjusted *p*-value for 1–10 parasites/ml: <0.001; adjusted *p*-value for >10 parasites:<0.001) (Table 3).

#### 3.3.2 Unadjusted multi-level associations with asymptomatic *Leishmania* infection

Bivariate analyses did not reveal a strong association between the odds of infection with *Leishmania* and any, household, or zone-level factor across all three villages (Table 3, supplementary data). Residing in a household with an external shelter (dase) was associated with a lower *L. donovani* infection rates in Dedevit (OR: 0.48, 95% CI: 0.25, 0.89), while having any animal led to an 86% increase in odds of infection among residents of Erdwayane (OR 1.86, 95% CI: 1.00, 3.44). In Salamo, the odds of *L.donovani* infections were significantly reduced among individuals in households with animal sheds (Dambe) outside the compound, as compared to those in households without animal sheds (OR 0.51, 95% CI: 0.26, 0.98) (Table 3, Supplementary data).

#### 3.3.3 Unadjusted multi-level associations with clinical disease

In Erdwayane and Salamo, the odds of developing clinical VL was significantly higher among males than among females (Erdwayane: OR: 2.04, 95% CI: 1.04, 4.02; Salamo: OR: 3.04, 95% CI: 1.57, 5.87) (Table 4, supplementary data). In Salamo (but not in Erdwayane or Dedevit) individuals residing in households with a stone fence were twice as likely to develop clinical disease as individuals residing in households without a stone fence (OR: 2.00, 95% CI: 1.09, 3.68).

#### 3.3.4 Adjusted multi-level associations

After adjustment for household-level (dase, stone fence, domestic animals) and eco-zone-level factors (vertisol and *Acacia* trees), significant differences between males and females were calculated. Males had 34% reduced odds of being infected asymptomatically (Table 4 A, OR: 0.66, 95% CI: 0.44, 0.97) yet were 2.8 times more likely to develop clinical VL (Table 4B, OR: 2.76, 95% CI: 1.52, 5.01).

**Table 4:** Adjusted Multilevel associations between *L. donovani* infections or VL, gender and environmental.

A- Multilevel analysis − Asymptomatic infection (PCR) positive	OR (95% CI)	P-value
Male sex	0.66 (0.44, 0.97)	0.035^**^
Dase	0.92 (0.49, 1.75)	0.803
Stone fence	0.76 (0.42, 1.39)	0.373
Any animal	0.85 (0.48, 1.49)	0.567
Vertisol	0.40 (0.10, 1.65)	0.206
Acacia	2.40 (0.82, 7.04)	0.111

B- Multilevel analysis − VL		
Male sex	2.76 (1.52, 5.01)	0.001^**^
Dase	1.25 (0.57, 2.71)	0.577
Stone fence	2.02 (0.88, 4.64)	0.099
Any animal	0.81 (0.42, 1.56)	0.519
Vertisol	0.26 (0.03, 2.72)	0.262
Acacia	1.02 (0.24, 4.43)	0.977

To explore the relationship between compositional variables (individual – or household level) and contextual variables (eco-zones-levels) versus number of *Ph. orientalis*, we included houses for which we had data on sand flies that were inhabited by individuals for whom clinical diagnostic data was available (32/39). Results showed that non-significant association (Table 5, Supplementary data). Nevertheless, the presence of urban elements and the using of better building quality materials, like corrugated iron roof and walls made of bricks and cement, tend to attract less sand flies, yet, no significant difference was found (K-W test, P = .843; Table 5, Supplementary data).

### 3.4 Spatial analysis

#### 3.4.1 *Ph. orientalis* population densities

**Fig. 5:**
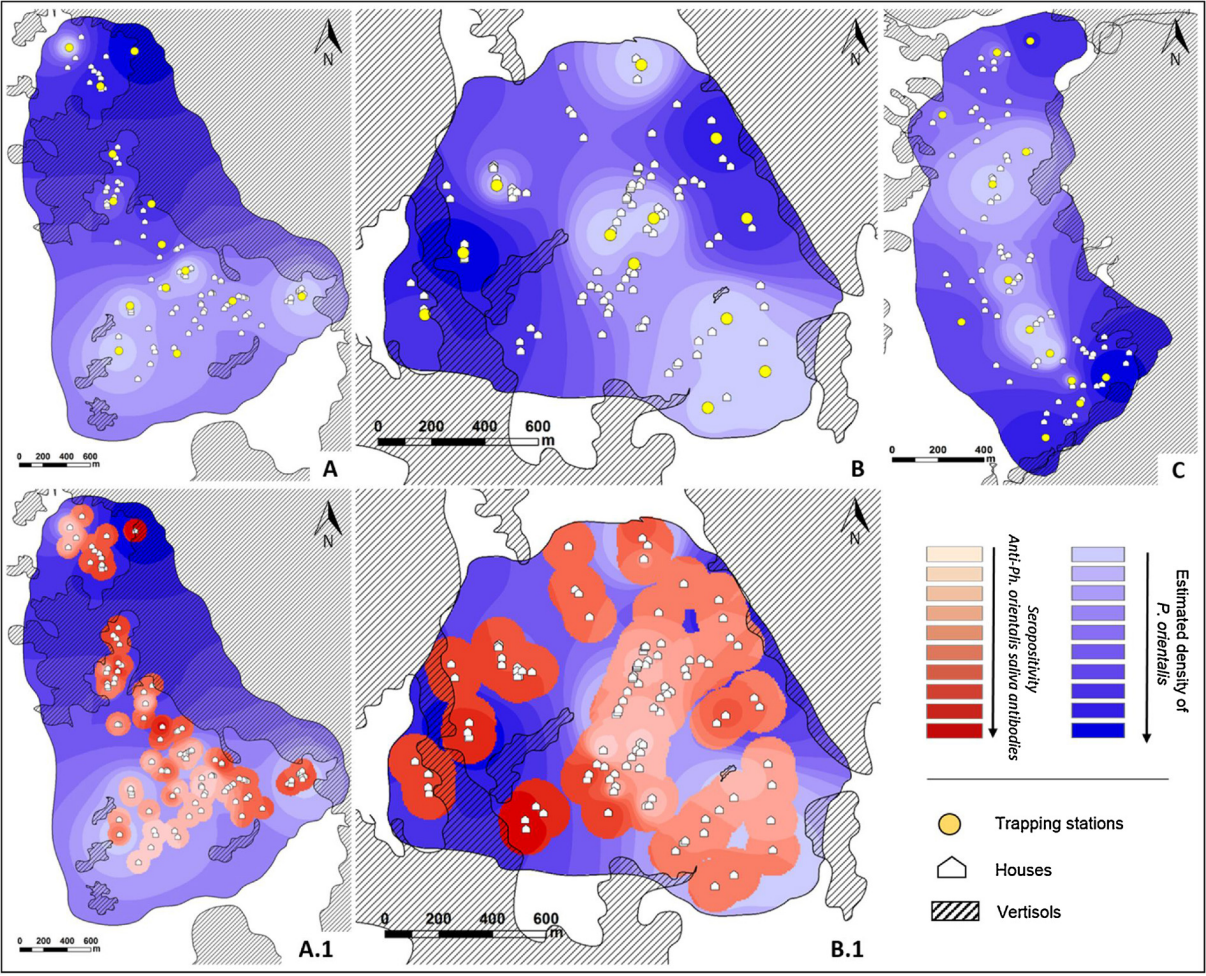
Maps of the three study villages showing sand fly densities derived by inverse distance weighted (IDW) interpolation model. **A** (Salamo), **B** (Dedevit) and **C** (Erdwayane) Blue shading represents the estimated density of *Ph. orientalis* based on TU yields. Interpolation results indicate denser sand fly populations (dark blue) close to vertisols (hatched). **A.1** (Salamo) and **B.1** (Dedevit) overlays in shades of red/pink depicting the intensity of seropositivity (optical density [OD]) to *Ph. orientalis* saliva represented as masked buffered circles (r = 100m) around each household. (For interpretation of the references to colour in this figure legend, the reader is referred to the web version of this article.)

To assess the densities of *Ph. orientalis* in different areas, trap yields were interpolated using Inverse Distance Weighting (IDW) and a surface interpolated map was constructed for each village. Areas with predicted high densities of *Ph. orientalis* were readily discernable (Fig. 5A–C, dark blue shading). The sand fly hotspots were almost exclusively congruent with vertisol fields located around the villages, or as restricted zones inside the village perimeters (Fig. 5 hatched areas) with scattered trees and shrubs (*Balanites* sp., *Acacia* sp. and/or *Zizyphus* sp.).

Linear regression was used to assess the effect of distance (Euclidian) from vertisols on the density of *Ph. orientalis*, where the distance was Ln transformed. In Salamo and Erdwayane the density of *Ph. orientalis* populations was negatively associated with the distance from vertisols (*F_(1.12)_* = 37.31, *P* = < 0.001; *R*^2^ = 0.75; *F_(1.11)_* = 8.99, *P* = < 0.001; *R*^2^ = 0.44, respectively). In Dedevit a significant negative association with distance from vertisols was established for females only (*F_(1.10)_* = 4.78, *P* = < 0.001; *R*^2^ = 0.32).

#### 3.4.2 Anti-sand fly saliva antibodies in volunteers’ blood

We also interpolated the anti-*Ph. orientalis* serological data (OD levels determined by ELISA) in serum samples obtained from volunteers in Salamo and Dedevit, the two villages with low numbers of *Ph. orientalis*. Using the IDW method we produced surface interpolated maps representing areas of intense exposure to sand fly bites (Fig. 5A.1 & B.1). The hotspots of anti-saliva sero-positivity largely overlapped the areas with dense *Ph. orientalis* populations close to vertisols. Linear regression predicted the relationship between exposure to sand fly bites (ODs, pink/red circles) and the abundance of *Ph. orientalis* sand flies (blue shading). A positive significant regression equation was found (Salamo: F_(1,111)_ = 34.535, p < 0.001, with an R^2^ of 0.237; Dedevit: F_(1,116)_ = 41.678, p < 0.001, with an R^2^ of 0.264)

#### 3.4.3 Distribution of VL cases and asymptomatic infections

**Fig. 6:**
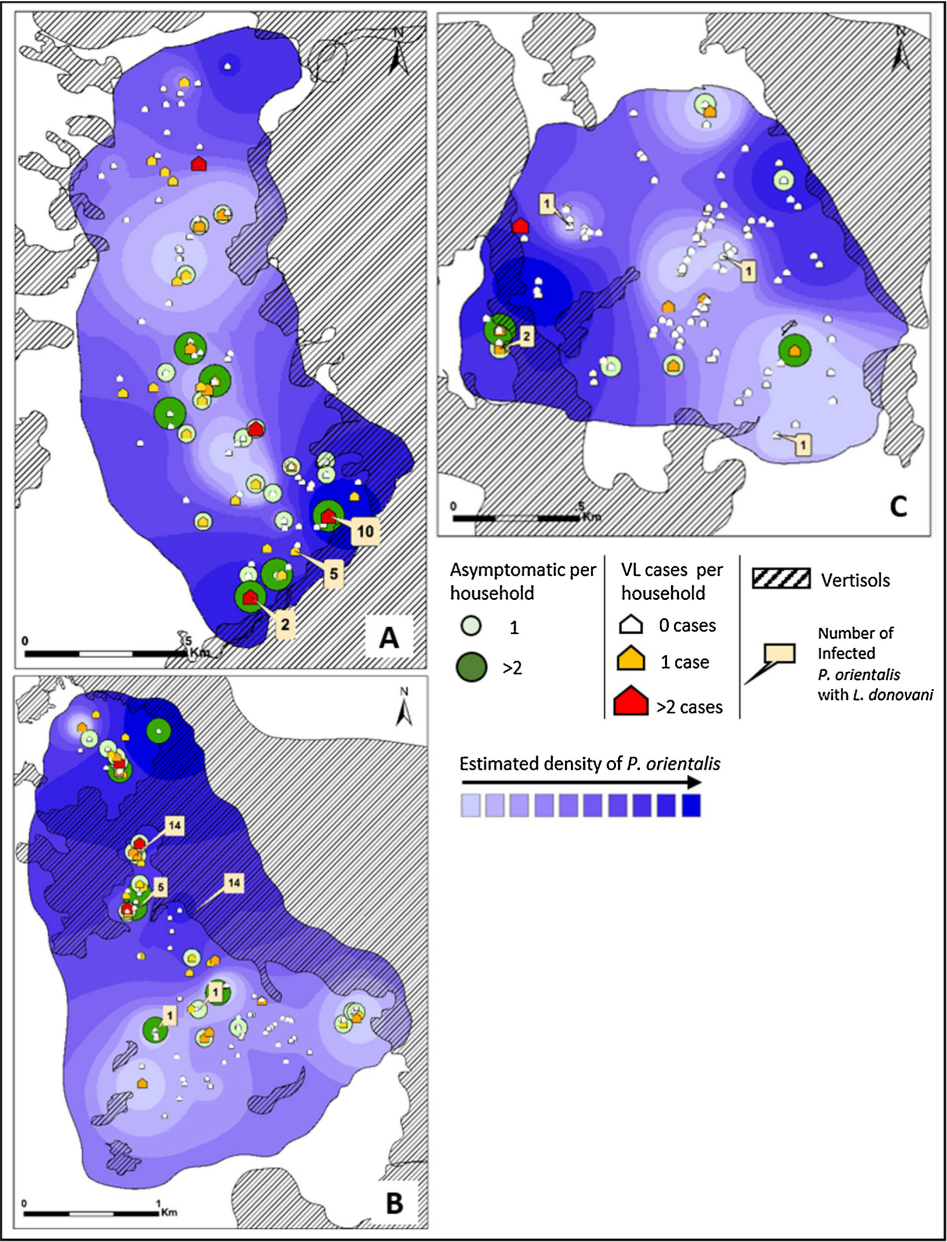
Spatial distribution of VL cases, asymptomatic infections and infected *Ph. orientalis* sand flies, plotted on interpolated surface maps showing the predictive distribution of *Ph. orientalis*. Maps were generated for each village, A) Erdwayane, B) Salamo and C) Dedevit. The distribution of VL cases is related to high density areas of *Ph. orientalis*. Likewise infected sand flies with *L. donovani* were more frequently found in households where more than two VL cases were found and close to vertisol areas.

We produced village maps showing the spatial distribution of households, VL cases (from 2007 to 2015) and asymptomatic PCR positive, individuals. These data were plotted on the maps representing the density of *Ph. orientalis*. Geographical; location and number of *L. donovani* infected *Ph. orientalis* were also marked on these maps (Fig. 6).

**Fig. 7:**
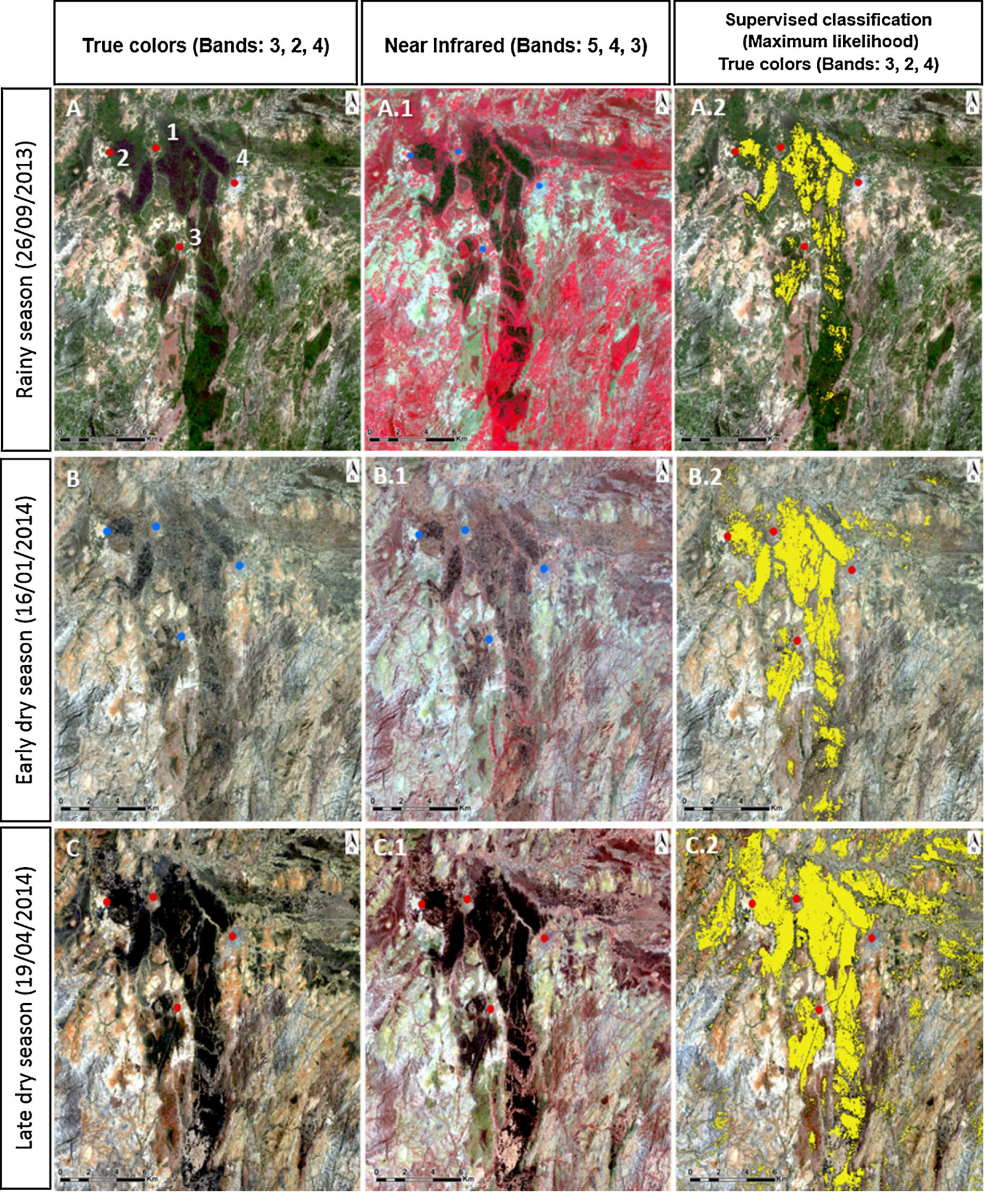
Satellite images (LANDSAT 8) of the study region showing: vertisol areas (true color images, pictures: A, B, C), the vegetation coverage (near infrared images, pictures: A.1, B.1, C.1) and vertisol areas classified images (Pictures: A.2, B.2, C.2) in different seasons. Photo A was acquired during the rainy season when the vegetation cover was dense, largely concealing the vertisols surface. Near infrared bands allows to visualize the vegetation in Red color, which accentuates the dense vegetation cover. Photos B were taken during the early dry season when seasonal vegetation had dried up but was still largely concealing the vertisol surface. Photos C were obtained during the late dry season when the vegetation coverage was relatively sparse revealing the dark vertisols. The three villages (1- Erdwayane, 2- Salamo, and 3-Dedevit) are clearly located within vertisols areas while the town of Sheraro (4) is located in sand stone area (prominently visible in 7A). In the classified images (pictures: A.2, B.2, C.2), vertisols were highlighted in yellow. In the early dry season (B.2), vertisols areas were still partially concealed by vegetation (natural and agricultural). In the late dry season (C.2), cracking vertisol areas become exposed, expanding as the vegetation cover receded posty harvest. Vertisols exposed to the sun, dry up and crack due to accelerated evaporation and subsequent shrinkage. (For interpretation of the references to colour in this figure legend, the reader is referred to the web version of this article.)

The maps show that VL cases and asymptomatic individuals appear not to be uniformly distributed, and houses with two or more VL cases and/or asymptomatic infected individuals, were more likely to be found relatively close to other households with infected individuals. Moreover, houses of patients and asymptomatic individuals were frequently located in areas with high abundance of *Ph. orientalis* sand flies. Sand flies infected with *L. donovani* were most frequently found near houses with infected individuals located a short distance from vertisols (Fig. 6).

In view of these observations it was somewhat surprising that, the prevalence of VL and asymptomatic infections was not significantly associated with the distance form vertisols, the *Ph. orientalis* population densities or the exposure of persons to their bites (data not shown).

### 3.5 Remote sensing analysis

LANDSAT 8 satellite pictures clearly show the vast expanses of vertisols within which our study sites were located (Fig. 7). Cracked vertisols become exposed during the dry season − black areas in true-color maps (Fig. 7A, B, and C). In the rainy season, wet soil swell, cracks seal-up and soil becomes partially concealed by annual vegetation (Fig. 7A1). The vegetation cover is easily discerned as red areas in infrared maps (Figs. 7A1, B1, and C1). To emphasize the phenomenon, vertisols were classified in yellow showing minimal areas in the rainy season (Fig. 7A2). Vast areas of vertisols are exposed as the dry season progresses (Figs. 7B2 and C2) because harvesting of agricultural crops and the drying up of seasonal vegetation, promote the soil’s visibility in satellite photography.

## 4 Discussion

Numerous epidemiological studies have been conducted aiming to identify risk factors that influence the transmission dynamics of VL. However, despite the significant amount of accumulated data, our understanding of the effects of anthropogenic and environmental factors on the transmission dynamics remain limited. We conducted studies in a relatively new focus of VL in the Tahtay Adiyabo district of northern Ethiopia, where VL presumably emerged due to mass influx of agricultural workers returning from the endemic regions of Humera and Metema. Military strife and resultant displacement of villagers along the border with Eritrea have probably contributed to this outbreak as well.

In 2011, we screened 4,900 volunteers from 11 villages for VL, asymptomatic infections and seropositivity to *L. donovani*. We found 14.3% (680/4,757) of the volunteers positive for *Leishmania* k-DNA and varying incidence of VL during the five preceding years (Table 1, Fig. 2) (Abbasi et al., 2013). Then we selected three of these villages, for further studies. Erdwayane and Salamo, with a relatively high number of VL cases and Dedevit with much fewer (Table 1, Fig. 2). Interestingly, the proportion of asymptomatic infections (qRT-kDNA/PCR) was markedly higher in Salamo compared with Erdwayane and Dedevit (Table 1).

Many infectious diseases affect males more than females. Such biases could potentially arise from physiological differences (e.g. sex hormones interacting with immune effectors) or from gender-related differences in exposure (Guerra-Silveira and Abad-Franch, 2013). Evidence in support of the former hypothesis was documented in hamsters infected with *Leishmania* (Travi et al., 2002). In humans, male-biased incidence of VL was described in studies in southern Ethiopia and Sudan (Ali and Ashford, 1994; Nackers et al., 2015). In our study too, a higher proportion of males developed VL (males: 69.2%, females: 30.7%), although more females were asymptomatically infected (10.9% of the males and: 13.3% of the females tested). Thus, our study design enabled us to determine that gender-related physiological traits and not behavioral differences between males and females were probably responsible for gender-specific differences in the incidence of VL.

On the other hand, behavioral parameters may have played a role in determining the distinct rates of infection and disease in the three villages (Table 1, Fig. 2). In Salamo and Erdwayane where VL incidence was high, most men are farmers working in the sorghum or sesame fields surrounding their villages and spending the nights in the village. In contrast, most men from Dedevit serve as soldiers in military bases located in the area, where they spend many nights on active duty outside the endemic village. These differences in behavioral profiles may explain the lowered incidence of VL in Dedevit.

The three villages are built on sand-stone outcrops surrounded by vertisols, long suspected as breeding and resting sites of sand flies in Sudan (Elnaiem et al., 1998; Elnaiem et al., 1999) and recently shown as such in Tahtey Adiabo (Moncaz et al., 2014). Despite the closeness of all three villages to vertisols, the densities of *Ph. orientalis* differed significantly with high densities in Salamo contrasting with significantly lower densities in Dedevit (Table 2). We attribute the lower densities in Dedevit to its more urban character with fewer domestic animals contrasting with the more rural landscape of Salamo and Erdwayane. Dedevit has asphalt roads and houses built closer together of commercial materials. We found a trend showing that lower numbers of *Ph. orientalis* were associated with houses built with commercial building materials. Nevertheless, we could not find a positive association between compositional variables and the density of *Ph. orientalis,* most probably because of the small number of traps used (Table 4).

Multi-level associations between compositional (individual or- household level) and contextual variables or factors (eco-zone level) that may potentially influence the transmission of VL did not reveal any significant relationships (Tables 2 & 3, Supplementary data). The highest rate of *L. donovani* infections was found in *Ph. orientalis* captured in [rural] Salamo and Erdwayane, while flies from [urban] Dedevit were seldom infected (Table 2). These observations may partially explain the relatively high rates of asymptomatic infections and VL among the residents of Salamo and Erdwayane compared with Dedevit (Figs. 2, 3).

We conducted restricted sampling of individuals who participated in the previous cohorts in the village of Salamo and Dedevit, where we found that, almost all of VL treated cases reported before and during the beginning of main cohort study (2011) remained seropisive by ELISA prospectively until 2015, and half of them were positive for *Leishmania* by kDNA-qRT/PCR.

In line with analysis obtained from the general cohort, performed in the 18 villages in the area, where individuals who were direct agglutination test (DAT) positive at baseline and re-sampled after a year, 29.1% (25/86) remained seropositive (Skrip unpublished). We found that a large number of healthy volunteers were also found to be seropositive by ELISA, demonstrating that there was a high proportion of the population, which had been exposed to *L. donovani* infections. Moreover, higher titers of anti-*L. donovani* antibodies were reported in the village of Salamo whereas in Dedevit titers were relatively low. We assume this was the case because all VL cases from Dedevit were relatively recent (since early 2010).

A high proportion of the *Ph. orientalis* females collected in inhabited compounds was found to have fed on bovine (79, 52%) or human (22, 14.7%) blood. The preponderance of bovine blood meals is undoubtedly attributable to the abundance of cows in peridomestic areas compared with other hosts (Gebresilassie et al., 2015a; Gebresilassie et al., 2015c). The abundance of cows close to houses may function as a zooprophylactic barrier, potentially reducing human-vector contact, or it may aggravate the risk of VL infection by attracting flies to houses and supplying them with ample protein-based nutrition required for egg production. Studies in Nepal showed that ownership or proximity of livestock was associated with significant protection from VL, whereas in India risk of VL increased for those living in close proximity to cattle (Bern et al., 2005; Singh et al., 2010). Our findings indicate that having any animal (cow, goat, chicken, camel, donkey, sheep, dog, and cat) increases the probability of being PCR positive for *L. donovani* in the village of Erdwayane (Table 2A, Supplementary data).

*Ph. orientalis* is the proven vector of *L. donovani* in the vast endemic foci of Sudan (Ashford et al., 1992; Elnaiem et al., 1998). The predominance of *Ph. orientalis* was reported in north-west Ethiopia too (Gebre-Michael et al., 2010; Gebresilassie et al., 2015c; Hailu et al., 1995). Our entomological studies demonstrated that *Ph. orientalis* was the most prevalent *Phlebotomus* spp., accounting for more than 93% overall and a relatively large proportion of the females were infected with *L. donovani* (Table 2) implicating this species as vector of *L. donovani*.

Previous studies in Northern Ethiopia have identified open vertisol fields as putative breeding grounds for sand flies, where larvae live in the deep vertisol cracks feeding on composting organic material (Moncaz et al., 2014). Here we identify cultivated vertisols as important sources of sand flies foraging in villages during the night (Fig. 5). GIS-based analysis allowed us to estimate the dispersion of *Ph. orientalis* from vertisols to households. Linear regression demonstrated a strong negative association between sand fly abundance and the distance from vertisols. Interpolated surface maps produced by converting point-based data (number of *Ph. orientalis* per TU) to surface data, allowed us to estimate the abundance of *Ph. orientalis* in different areas within each village (Fig. 5). In support of this assertion, we also describe a marked periodicity in the activity patterns of *Ph. orientalis*, with a peak between 21:00–22:30 in open field and a delayed peak of activity 22:30-00:00, in peridomestic habitat. These finding were also reported in previous studies conducted in Northern Ethiopia (Gebresilassie et al., 2015b; Lemma et al., 2014). We interpret this delay to imply that sand flies foraging in the villages had arrived from the adjacent vertisol fields (Fig. 4).

Furthermore, in additional GIS-based analyses, we measured the levels of sero-positivity (optical density [OD] by ELISA) to-*Ph. orientalis* saliva as an indicator of exposure to biting sand flies. Careful examination of the resultant surface maps indicates higher levels of α-*Ph. orientalis* saliva antibodies in volunteers living close to vertisols where sand fly populations are more dense (Fig. 5).

Our GIS-based maps, indicate that VL cases were frequently clustered, with more than one case per house or groups of houses (Fig. 6). In addition, houses of VL patients as well as asymptomatic infected persons were frequently located relatively close to vertisols where sand fly populations were denser (Fig. 6). Hence, proximity to vertisols comprises a risk factor for contracting VL. Taken together, these findings support the hypothesis that, transmission of *L. donovani* probably occurs anthroponotically (humans serve as parasite reservoir) in or close to households rather than in the fields. In conclusion, sand flies breeding in vertisols, are attracted into villages to feed blood on domestic animals and humans. They are infected with *L. donovani* as they feed on infected humans (VL patients and asymptomatic cases) and transmit the parasites to naïve individuals living in proximity during subsequent blood meals. Thus, living close to vertisols comprises the single most important risk factor for contracting infections with *L. donovani,* in foci where the parasite is transmitted by *Ph. orientalis.*
