# Supplementary material for: A fine scale eco-epidemiological study on endemic visceral leishmaniasis in north ethiopian villages
Source: Acta Trop. 2018 Jul;183:64–77. doi: 10.1016/j.actatropica.2018.04.005 (PMC5956276; doi:10.1016/j.actatropica.2018.04.005)
Supplement: Supplementary file 1 [file mmc1.docx]

**Supplementary Data**

| **Table 1 (Supp. data)** - Relationship between anti-*L. donovani* serology (ELISA) and infection (qRT-kDNA/PCR) positivity in volunteers from endemic villages | | | | | | |
| --- | --- | --- | --- | --- | --- | --- |
| Dedevit + Salamo | | **qPCR Finger Prick** | | | | **Total** |
|  |  | **VL Treated cases** | | **Healthy** | |  |
|  |  | **Positive** | **Negative** | **Positive** | **Negative** |  |
| **ELISA** | **Positive** | 20 (15.8%) | 20 (15.8%) | 10 (7.9%) | 35 (27.7%) | 85 |
|  | **Negative** | 1 (0.7%) | 3 (2.3%) | 4 (3.1%) | 33 (26.1%) | 41 |
| **Total** |  | 21 | 23 | 14 | 68 | 126 |

| **Table 2 (Supp. Data) –** Relative abundance and fauna of sand flies collected from the three sampling villages (2013) | | | |
| --- | --- | --- | --- |
| **Sand fly species** | **♂/♀** | **Total** | **Frequency (%)** |
| *Phlebotomus orientalis* | 1,444/ 887 | 2,331 | 93.9% |
| *P. duboscqi* | 55/ 34 | 89 | 3.6% |
| *P. bergeroti* | 28/ 19 | 47 | 1.9% |
| *P. rodhaini* | 1/ 6 | 7 | 0.3% |
| *P. alexandri* | 0/ 4 | 4 | 0.2% |
| *P. papatasi* | 0/ 4 | 4 | 0.2% |
| *P. martini* | 1/ 0 | 1 | 0.0% |
| *Sergentomyia africana* | 1,157/ 2250 | 3,407 | 71.1% |
| *S. antennata* | 167/ 276 | 443 | 9.2% |
| *S. schwetzi* | 157/ 248 | 405 | 8.5% |
| *S. clydei* | 67/ 108 | 175 | 3.7% |
| *S. squamiplueris* | 47/ 55 | 102 | 2.1% |
| *S. bedfordi* | 39/ 47 | 86 | 1.8% |
| *S. adleri* | 35/ 49 | 84 | 1.8% |
| *S. christophersi* | 15/ 40 | 55 | 1.1% |
| *S. calcarata* | 9/ 30 | 30 | 0.6% |
| *S. dubia* | 1/ 4 | 5 | 0.1% |
| Percentages of *Phlebotomus* were calculated over the total number of sand flies captured (since they were separated from the whole collection). However, percentages of *Sergentomyia* were calculated over the total number of sand flies dissected which were no *Phlebotomus* (17,194 total Sergentomyia, of these, 4,792 were dissected, representing the 28% of the collection). | | | |

| **Table 3 (Supp. data) –** Bivariate analyses assessing relationships between multilevel factors as fixed effects and PCR positivity (asymptomatic infections) by village. | | | |
| --- | --- | --- | --- |
| **PCR Positivity** | **Erdwayane** | **Salamo** | **Dedevit** |
| Male sex | 0.86 (0.49, 1.48) | 1.11 (0.75, 1.66) | 0.65 (0.39, 1.10) |
| Dase (Resting shelter) | 1.23 (0.65, 2.33) | 1.07 (0.67, 1.73) | 0.48 (0.25, 0.89) ** |
| Stone fence | 0.61 (0.34, 1.08) | 0.87 (0.58, 1.29) | N/A |
| Dambe (Animal shed) |  |  |  |
| None | ref | ref | ref |
| Inside compound | 1.99 (1.10, 3.59) | 0.87 (0.55, 1.36) | 0.69 (0.38, 1.24) |
| Outside compound | 0.98 (0.32, 3.04) | 0.51 (0.26, 0.98) ** | 1.21 (0.56, 2.62) |
| Any animal | 1.86 (1.00, 3.44) ** | 1.16 (0.66, 2.05) | 0.30 (0.14, 0.66) |
| Vertisol | N/A | N/A | 0.73 (0.33, 1.57) |
| Acacia | N/A | N/A | 0.98 (0.55, 1.73) |
| OR (95% CI)  ** Statistically significant at p<0.05  Unadjusted multi-level associations with asymptomatic Leishmania infection | | | |

| **Table 4 (supp. data) -**  Bivariate analyses assessing relationship between multilevel factors as fixed effects and clinical VL cases by village | | | |
| --- | --- | --- | --- |
| **VL Clinical Case** | **Erdwayane** | **Salamo** | **Dedevit** |
| Male sex | 2.04 (1.04, 4.02) ** | 3.04 (1.57, 5.87) ** | 4.25 (0.88, 20.70) |
| Dase (Resting shelter) | 1.89 (0.81, 4.38) | 2.07 (0.86, 4.98) | 0.66 (0.14, 3.26) |
| Stone fence | 1.82 (0.78, 4.23) | 2.00 (1.09, 3.68) ** | N/A |
| Dambe (Animal Shed) |  |  |  |
| None | ref | ref | ref |
| Inside compound | 1.21 (0.63, 2.32) | 0.63 (0.32, 1.23) | 0.21 (0.04, 1.18) |
| Outside compound | N/A | 0.83 (0.35, 1.95) | 1.45 (0.32, 6.69) |
| Any animal | 0.54 (0.24, 1.21) | 0.90 (0.38, 2.15) | 0.52 (0.06, 4.39) |
| Vertisol | N/A | N/A | 0.91 (0.11, 7.37) |
| Acacia | N/A | N/A | 2.33 (0.62, 8.83) |
| OR (95% CI)  ** Statistically significant at p<0.05  Unadjusted multi-level associations with clinical disease. | | | |

| **Table 5 (Supp. data)** | | | |
| --- | --- | --- | --- |
|  | **Number of households (n=37)** | **Median of P. orientalis (IQI)** | **P-value**** |
| ***Phlebotomus orientalis* abundance Vs. Clinical variables** |  |  |  |
| At least one PCR+ household member (>0 parasites per ml blood) |  |  | 0.462 |
| No | 16 | 15.3 (4.5, 48.0) |  |
| Yes | 21 | 10.0 (4.0, 22.7) |  |
| At least one PCR+ household member (>10 parasites per ml blood) |  |  | 0.810 |
| No | 29 | 10.0 (4.0, 24.0) |  |
| Yes | 8 | 11.7 (7.2, 28.8) |  |
| ***Phlebotomus orientalis* Vs. Domestic variables** |  |  |  |
| Dase (outside shelter) |  |  | 0.798 |
| No | 10 | 10.0 (5.5, 29.0) |  |
| Yes | 27 | 13.3 (3.3, 24.0) |  |
| Dambe (Animal shed) |  |  | 0.655 |
| None | 12 | 10.0 (3.5, 19.5) |  |
| Inside compound | 19 | 8.0 (4.3, 28.5) |  |
| Outside compound | 6 | 20.0 (9.8, 29.7) |  |
| Any animal (camel, donkey, goat, sheep, dog, cat) |  |  | 0.843 |
| No | 10 | 19.8 (4.3, 24.0) |  |
| Yes | 12 | 11.7 (5.0, 29.8) |  |
| Stone fence |  |  | 0.174 |
| No | 7 | 19.3 (7.0, 110.7) |  |
| Yes | 30 | 10.0 (3.0, 23.8) |  |
| Tin roof |  |  | 0.387 |
| No | 6 | 23.0 (6.8, 50.9) |  |
| Yes | 31 | 10.0 (4.0, 23.5) |  |
| ** P-value calculated using the Kruskal Wallis test  Data is presented as median (IQR = Interquartile Intervals). | | | |
